# Gemcitabine activates the Hippo signaling pathway and suppresses tumor growth by stabilizing large tumor suppressor kinase 2 through the hypoxia‐inducible factor 1‐alpha/ubiquitin protein ligase E3 component N‐recognin 5 axis

**DOI:** 10.1002/ccs3.70085

**Published:** 2026-06-17

**Authors:** Jin Zhang, Lixia Xu, Kai Guo, Weijia Dong, Xin Dai, Donghua Gu

**Affiliations:** ^1^ Department of Pathology Suzhou Science & Technology Town Hospital Suzhou China; ^2^ Department of Pathology Huzhou Central Hospital Huzhou China; ^3^ Department of Pathology School of Medicine Huzhou University Huzhou China

**Keywords:** gemcitabine, HIF1A, Hippo signaling pathway, ovarian cancer, UBR5

## Abstract

To investigate the molecular mechanism by which gemcitabine (GEM) inhibits ovarian cancer (OC) progression, focusing on the hypoxia‐inducible factor 1‐alpha (HIF1A)/UBR5/LATS2 axis and Hippo pathway. Bioinformatics analysis of gene expression omnibus datasets identified HIF1A‐associated modules. Transcriptional regulation of UBR5 by HIF1A was validated via ChIP‐PCR and dual‐luciferase assays. Anti‐tumor effects of GEM were assessed in OC cell lines and an orthotopic mouse model using functional assays (CCK‐8, transwell), Western blot, Co‐IP, and ubiquitination analysis. HIF1A transcriptionally upregulates UBR5 in OC. GEM downregulated the HIF1A/UBR5 axis, suppressing OC cell proliferation, migration, and invasion. Mechanistically, UBR5 promoted LATS2 ubiquitination and degradation. GEM inhibited this interaction, stabilizing LATS2, activating the Hippo pathway, and suppressing downstream YAP1/FGFR1 signaling. In vivo, GEM inhibited tumor growth and downregulated HIF1A, UBR5, and FGFR1. GEM suppresses OC progression by targeting the HIF1A/UBR5 axis, which subsequently stabilizes LATS2, activates the Hippo pathway, and inhibits YAP1/FGFR1 signaling, revealing a novel therapeutic mechanism.

## Graphical abstract

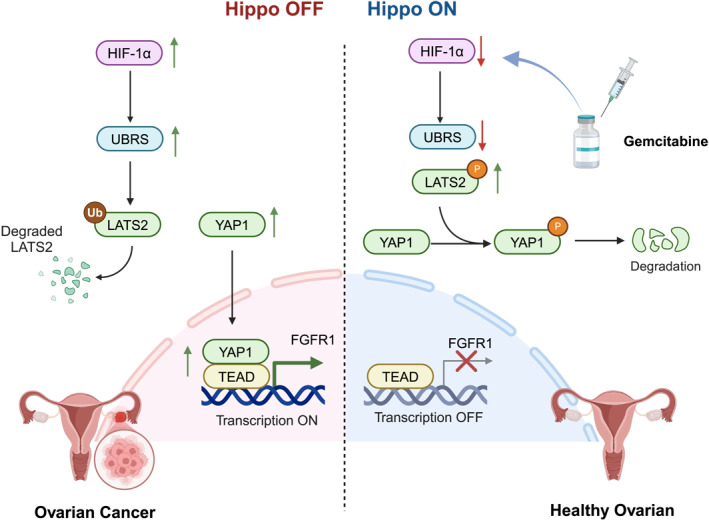

## 1 INTRODUCTION

Ovarian cancer (OC) remains one of the most lethal malignancies within the female reproductive system and is a global focal point for cancer research.^1^, ^2^ Because early‐stage OC often lacks distinctive clinical symptoms,^3^ most patients are diagnosed only after the disease has progressed to an advanced stage.^4^ Global cancer statistics indicate that the 5‐year survival rate of OC is markedly lower than that of other gynecological cancers.^5^ Current first‐line treatment strategies mainly involve surgical resection, radiotherapy, chemotherapy, and targeted therapy.^5^ However, the effectiveness of these interventions is often limited due to the complexity of OC cells and their high resistance to chemotherapy agents.^6^ Additionally, a high recurrence rate poses a significant challenge in treating OC. Consequently, developing new therapeutic strategies and identifying novel treatment targets is critically urgent.

Hypoxia‐inducible factor 1‐alpha (HIF1A) plays a vital role in various cancers by regulating the oxygenation status within the tumor microenvironment.^7^, ^8^ In OC, elevated HIF1A expression is strongly linked to enhanced tumor aggressiveness and unfavorable prognosis.^9^ Studies indicate that HIF1A promotes tumor angiogenesis and cellular proliferation, increasing tumor treatment resistance.^10^ Additionally, UBR5, an E3 ubiquitin‐protein ligase, is essential for regulating protein ubiquitination and degradation.^11^ Overexpression of UBR5 in OC cells correlates with poor prognosis^12^ and impacts tumor cell survival and proliferation by regulating the stability and function of tumor‐related proteins through a ubiquitin‐dependent pathway.^13^ Therefore, HIF1A and UBR5 are key focal points in researching OC's pathological mechanisms and therapeutic targets. It is worth noting that under normoxic conditions, HIF1A undergoes hydroxylation by prolyl hydroxylase domain enzymes, followed by recognition and ubiquitination by the VHL E3 ubiquitin ligase, ultimately leading to its degradation via the proteasome pathway.^14^ UBR5, also an E3 ubiquitin ligase, has been shown to mediate the ubiquitination and degradation of various proteins.^11^ Given the high co‐expression of HIF1A and UBR5 across various tumor types and their shared involvement in hypoxia‐related signaling pathways, it was hypothesized that HIF1A may transcriptionally regulate UBR5; however, this regulatory relationship has not yet been experimentally confirmed.

The Hippo signaling pathway is a crucial regulator of cellular proliferation and apoptosis, with increasing research in tumor biology in recent years. In OC, aberrant activation of the Hippo pathway is associated with enhanced cellular proliferation, migration, and invasion capabilities.^15^ Core components of this pathway, such as YAP/TAZ, can drive the expression of pro‐growth and anti‐apoptotic genes through interaction with the TEAD family of transcription factors.^16^, ^17^ Modulating the activity of the Hippo pathway could serve as a potential strategy to control the progression of OC.^16^ Tao et al. demonstrated that HIF1A influences the development of colorectal cancer by regulating LATS2 expression.^18^ Zhang et al. demonstrated that HIF1A participates in hypertension and vascular remodeling through regulation of the Hippo signaling pathway.^19^ Similarly, Yang et al. reported that the HIF1A/Hippo signaling axis regulates cell proliferation and apoptosis.^20^ However, the specific contribution of HIF1A/Hippo signaling to OC onset and progression remains unclear. Meanwhile, several E3 ubiquitin ligases have been reported to be key regulators of Hippo pathway activity,^21^, ^22^, ^23^ yet it has not been confirmed whether UBR5 is involved in the regulation of this pathway.

Gemcitabine (GEM), a widely used nucleoside chemotherapeutic agent, is primarily employed in treating various solid tumors, such as pancreatic and breast cancer.^24^ In OC, GEM inhibits tumor cell proliferation by interfering with DNA replication and repair mechanisms.^25^ Earlier studies indicate that GEM may produce its antitumor activity by regulating HIF1A expression,^26^ although the precise molecular mechanisms underlying its interaction with HIF1A remain unclear. Therefore, elucidating the precise molecular mechanisms by which GEM inhibits OC progression could provide valuable therapeutic targets and potentially lead to more effective personalized treatments, ultimately improving clinical outcomes and patient survival.

Based on transcriptomic data from OC and weighted gene co‐expression network analysis (WGCNA), we hypothesize that GEM may suppress HIF1A expression and influence the Hippo signaling pathway via UBR5‐mediated ubiquitination. This regulation may further affect FGFR1 and YAP1 levels, ultimately inhibiting the growth, migration, and invasion of OC cells. To verify this hypothesis, we aim to elucidate the molecular mechanisms through which GEM modulates the HIF1A/UBR5 axis and assess its potential therapeutic value in OC. This study is expected to provide novel insights into the anti‐OC mechanisms of GEM and contribute to the development of more effective personalized treatment strategies, offering potential benefits for patients worldwide.

## 2 MATERIALS AND METHODS

### 2.1 Public data download

OC‐related datasets were retrieved from the gene expression omnibus (GEO) database, including 16 tumor epithelial samples (GSM1314228–GSM1314243) and 6 normal epithelial controls (GSM1314222–GSM1314227). RNA‐seq data in TPM format, uniformly processed through the Toil pipeline from TCGA and GTEx, were obtained from UCSC XENA (https://xenabrowser.net/datapages/). From this dataset, RNA‐seq data corresponding to ovarian serous cystadenocarcinoma were extracted, including 376 tumor tissue samples from TCGA and 180 normal tissue samples from GTEx. Ethical approval or informed consent was not required because these data were obtained from a public database.

### 2.2 Differential expression analysis

Using the GEO high‐throughput transcriptomic datasets, differentially expressed genes (DEGs) were identified using the *R* package Limma (linear models for microarray data) with the criteria of |log_2_FoldChange| > 1 and *p* < 0.05. Volcano plots for visualizing DEGs were generated using “ggplot2.” All analyses were conducted in *R* version 4.3.1 (*R* Foundation for Statistical Computing).

### 2.3 Functional enrichment analysis

For the gene set functional enrichment analysis, we utilized the gene ontology (GO) annotations from the *R* package org.Hs.eg.db (version 3.1.0) as a background to map genes to the background dataset. Enrichment analysis was conducted using the *R* package clusterProfiler (version 3.14.3) to determine the enriched gene sets. For pathway analysis, we accessed the latest Kyoto encyclopedia of genes and genomes (KEGG) pathway gene annotations using the KEGG REST API (https://www.kegg.jp/kegg/rest/keggapi.html). Using the same *R* package, clusterProfiler, we performed enrichment analysis with a minimum gene set size of 5 and a maximum of 5000, using a *p*‐value of <0.05 and an FDR of <0.25. Data visualization was carried out using the ggplot2 package.

### 2.4 WGCNA

Based on gene expression profiles, the median absolute deviation (MAD) was calculated for each gene, and the lowest 50% of genes ranked by MAD were removed. Outlier genes and samples were further filtered using the goodSamplesGenes function in the *R* package WGCNA. A scale‐free co‐expression network was then constructed with WGCNA, with the minimum module size set to 30 and the sensitivity parameter set to 3. Modules with a distance below 0.25 were merged, resulting in 38 co‐expression modules. The gray module represented genes that could not be assigned to any specific module.

Tumor samples were stratified into high‐ and low‐expression groups according to HIF1A expression levels. Pearson correlation analysis (*p* < 0.05) was performed to assess the associations between these groups and the identified modules. Genes within the most significantly correlated module were defined as HIF1A‐related genes and used for subsequent analyses.

### 2.5 Protein–protein interaction (PPI) network analysis

A PPI network was constructed for the selected genes using the STRING database and visualized with Cytoscape software (version 3.7.2).

### 2.6 Receiver operating characteristic (ROC) curve analysis

The diagnostic value of HIF1A and UBR5 in OC was assessed using ROC curve analysis with the pROC package in *R* (version 1.18.0). The curves were plotted, and the area under the curve (AUC) values was calculated. In this analysis, tissue type (tumor vs. normal) was used as the categorical variable, whereas gene expression levels served as continuous predictive variables. All plots were generated using the ggplot2 package with a unified style applied via the theme_bw function.

### 2.7 Group‐based expression analysis and correlation analysis

To determine whether UBR5 expression is regulated by HIF1A, all samples in the TCGA‐OV dataset were divided into high and low HIF1A expression groups based on the median expression value. The expression levels of UBR5, YAP1, and FGFR1 were then compared between the two groups using the Wilcoxon rank‐sum test, and the results were visualized with box plots generated by the ggpubr *R* package (v0.6.0). The correlation between HIF1A and UBR5, YAP1, and FGFR1 expression levels was assessed using Pearson's correlation coefficient. The cor. test function was used to calculate the correlation coefficient (*r*) and corresponding *p*‐value. Expression data from TCGA‐OV samples were employed for this analysis, with all values normalized as log 2 (FPKM + 1). Scatter plots displaying the correlation were created using ggplot2, with linear regression trend lines shaded to indicate 95% confidence intervals.

### 2.8 JASPAR sequence prediction analysis

The prediction of HIF1A binding sites was performed using the JASPAR database (http://jaspar.genereg.net). Position frequency matrices (MA1106.1 and MA1106.2) for HIF1A were used to scan the human UBR5 promoter region (−2000 bp to +200 bp), and potential hypoxia‐response element (HRE) sequences were identified using the default threshold of 85%.

### 2.9 Cell culture, lentiviral infection, and grouping

Human OC cell lines SKOV3 (HTB‐77, ATCC) and OVCAR‐3 (HTB‐161, ATCC) were obtained from ATCC. According to ATCC records, SKOV3 originates from ovarian adenocarcinoma tissue of a 64‐year‐old female patient (RRID: CVCL_0532), whereas OVCAR‐3 was established from malignant ascites of a 60‐year‐old female patient with progressive ovarian adenocarcinoma (RRID: CVCL_0465). Before experiments, both cell lines were verified by STR profiling and confirmed to be mycoplasma‐free using a commercial detection kit. Cells within 20 passages after thawing were used for all experiments.

SKOV3 and OVCAR‐3 cells were maintained in high‐glucose DMEM (C0891, Beyotime) containing 10% fetal bovine serum (FBS; 12483020, Gibco), 100 U/mL penicillin, and 100 μg/mL streptomycin (C0222, Beyotime). Cells were incubated at 37°C in a humidified atmosphere with 5% CO_2_. Hypoxic culture was performed under 1% O_2_ conditions.^27^

For GEM treatment, SKOV3 and OVCAR‐3 cells were seeded into 6‐well plates and allowed to attach for 4 h. Cells were then treated with GEM (G6423, Merck) at 5, 10, or 20 μM by adding 200 μL GEM solution to each well, whereas control wells received PBS. Cells were collected after 0, 24, or 48 h for subsequent assays.

Recombinant lentiviruses and corresponding negative control vectors were generated by Shanghai GeneChem Co., Ltd. SKOV3 and OVCAR‐3 cells in the logarithmic growth phase were collected and resuspended at 5 × 10^4^ cells/mL. The cell suspension was seeded into 6‐well plates at 2 mL/well and cultured overnight at 37°C. Lentiviruses for gene overexpression or knockdown were then added at a final concentration of 1 × 10^8^ TU/mL. After 48 h of infection, the cells were used for further experiments.

Experimental grouping for GEM concentration gradient to inhibit HIF1A and UBR5: PBS group: SKOV3 or OVCAR‐3 cells were treated with PBS for 0, 24, and 48h. GEM 5 μM group: SKOV3 or OVCAR‐3 cells were treated with 5 μM GEM for 0, 24, and 48h. GEM 10 μM group: SKOV3 or OVCAR‐3 cells were treated with 10 μM GEM for 0, 24, and 48 h. GEM 20 μM group: SKOV3 or OVCAR‐3 cells were treated with 20 μM GEM for 0, 24, and 48 h.

Experimental grouping for recovery experiments on HIF1A and UBR5 inhibition by GEM: PBS group: SKOV3 or OVCAR‐3 cells were treated with PBS for 48h. GEM 20 μM (48 h) group: SKOV3 or OVCAR‐3 cells were treated with 20 μM GEM for 48h. GEM 20 μM (48 h) + oe‐NC group: SKOV3 or OVCAR‐3 cells were infected with an overexpression control lentivirus and treated with 20 μM GEM for 48h. GEM 20 μM (48 h) + oe‐HIF1α group: SKOV3 or OVCAR‐3 cells were infected with a lentivirus overexpressing HIF1α and treated with 20 μM GEM for 48 h. GEM 20 μM (48 h) + oe‐UBR5 group: SKOV3 or OVCAR‐3 cells were infected with a lentivirus overexpressing UBR5 and treated with 20 μM GEM for 48h.

Experimental grouping for regulating UBR5 by GEM via inhibition of HIF1A expression: PBS group: SKOV3 or OVCAR‐3 cells were treated with PBS for 48 h. GEM group: SKOV3 or OVCAR‐3 cells were treated with 20 μM GEM for 48 h. GEM + oe‐NC group: SKOV3 or OVCAR‐3 cells infected with an overexpression control lentivirus were treated with 20 μM GEM for 48 h. GEM + oe‐HIF1α group: SKOV3 or OVCAR‐3 cells infected with a lentivirus overexpressing HIF1A were treated with 20 μM GEM for 48 h. GEM + oe‐HIF1α + shNC group: SKOV3 or OVCAR‐3 cells infected with lentiviruses overexpressing HIF1A and a knockdown control were treated with 20 μM GEM for 48 h. GEM + oe‐HIF1α + shUBR5 group: SKOV3 or OVCAR‐3 cells infected with lentiviruses overexpressing HIF1A and knocking down UBR5 were treated with 20 μM GEM for 48 h.

Experimental groups for regulating LATS2 ubiquitination by GEM via UBR5: PBS group: SKOV3 or OVCAR‐3 cells were treated with PBS for 48 h. GEM group: SKOV3 or OVCAR‐3 cells were treated with 20 μM GEM for 48 h. GEM + oe‐NC group: SKOV3 or OVCAR‐3 cells, infected with an overexpression control lentivirus, were treated with 20 μM GEM for 48 h. GEM + oe‐UBR5 group: SKOV3 or OVCAR‐3 cells, infected with a lentivirus overexpressing UBR5, were treated with 20 μM GEM for 48 h.

Experimental groups for UBR5 regulation of the Hippo signaling pathway: GEM + oe‐NC group: SKOV3 or OVCAR‐3 cells, infected with an overexpression control lentivirus, were treated with 20 μM GEM for 48 h. GEM + oe‐YAP1 group: SKOV3 or OVCAR‐3 cells, infected with a lentivirus overexpressing YAP1, were treated with 20 μM GEM for 48 h. GEM + oe‐FGFR1 group: SKOV3 or OVCAR‐3 cells, infected with a lentivirus overexpressing FGFR1, were treated with 20 μM GEM for 48 h. GEM + oe‐YAP1 + oe‐FGFR1 group: SKOV3 or OVCAR‐3 cells, infected with lentiviruses overexpressing FGFR1 and YAP1, were treated with 20 μM GEM for 48 h.

The shNC sequence was 5′‐TTCTCCGAACGTGTCACGT‐3′; and the shUBR5 sequence was 5′‐TTGGAACAGGCTACTATTAAA‐3′.

### 2.10 Western blot (WB) analysis

Cellular proteins were prepared by lysing the collected cells in RIPA buffer supplemented with PMSF (P0013B, Beyotime, China). The protein content of each sample was quantified using a BCA assay kit (23225, Thermo Fisher Scientific, USA). Equal amounts of protein, ranging from 30 to 50 μg, were combined with 2 × SDS sample buffer and heated at 95°C for 5 min before electrophoretic separation by SDS‐PAGE. Proteins were then transferred onto PVDF membranes (0.45 μm, Millipore, USA) under wet transfer conditions. Membranes were blocked with 5% non‐fat milk at room temperature for 1 h followed by overnight incubation at 4°C with primary antibodies. The following primary antibodies were used as internal controls: HIF1A (A22041, ABclonal, China, 1:1000); UBR5 (A13816, ABclonal, China, 1:1000); FGFR1 (A21219, ABclonal, China, 1:1000); LATS2 (20276‐1‐AP, Proteintech, USA, 1:1000); YAP1 (ab205270, Abcam, UK, 1:1000); p‐YAP1 (ab62751, Abcam, UK, 1:1000); Ubiquitin (ab7780, Abcam, UK, 1:1000); and *β*‐actin (ab8226, Abcam, UK, 1:5000). Membranes were washed three times with TBST (10 min each), then incubated with HRP‐conjugated secondary antibody goat anti‐rabbit IgG (ab97051, Abcam, UK, 1:2000) at room temperature for 1 h. After additional washes, signal development was performed using the Pierce ECL WB Substrate (32209, Thermo Fisher Scientific, USA), and images were acquired with the ChemiDoc XRS + imaging system (Bio‐Rad, USA).

### 2.11 Ubiquitination assay

To assess the ubiquitination level of LATS2 mediated by UBR5, cells were pretreated with MG132 (20 μM, HY‐13259, MedChemExpress, USA) for 4 h to inhibit proteasomal degradation. Cells were then harvested and lysed on ice for 30 min using IP lysis buffer containing protease inhibitors and 10 mM N‐ethylmaleimide. Lysates were clarified by centrifugation at 12,000 × g. Dynabeads Protein G (10003D, Thermo Fisher Scientific, USA) pre‐coated with either anti‐UBR5 antibody (sc‐271499, Santa Cruz, USA) or anti‐LATS2 antibody (20276‐1‐AP, Proteintech, USA) were incubated with the clarified lysates at room temperature for 1 h, followed by rotation at 4°C for 2–4 h. Following four washes with pre‐chilled IP lysis buffer, the precipitated immune complexes were eluted in 2 × SDS loading buffer by boiling at 95°C for 5 min. The samples were then resolved by SDS‐PAGE and analyzed by WB. Ubiquitination signals were examined using an anti‐ubiquitin antibody (ab7780, Abcam, UK).

### 2.12 Cell counting kit‐8 (CCK‐8) assay

SKOV3 and OVCAR‐3 cells were plated in 96‐well plates at a density of 4 × 10^3^ cells per well in 100 μL of complete culture medium. After allowing cells to adhere for 24 h, GEM was applied according to the designated experimental groups. At 24 h and 48 h following treatment, 10 μL of CCK‐8 reagent (C0037, Beyotime, China) was added to each well, and the plates were incubated at 37°C for 1–2 h. Cell viability was assessed by measuring absorbance at 450 nm using a microplate reader. Background values were corrected by subtracting readings from blank wells containing only culture medium and CCK‐8 solution. The resulting data were then used for statistical analysis.

### 2.13 Transwell assay

Cell invasion was evaluated using 24‐well transwell inserts with 8 μm pores (353097, BD Biosciences). Before cell seeding, the upper inserts were coated with Matrigel (356234, BD Biosciences) diluted at 1:8 and incubated at 37°C for 1 h to promote gel formation. After the indicated treatments, SKOV3 and OVCAR‐3 cells were harvested and suspended in serum‐free medium. A total of 2 × 10^5^ cells were added to each upper chamber, whereas complete medium supplemented with 10% FBS was placed in the lower chamber as the chemoattractant. Following incubation for 24 h at 37°C with 5% CO_2_, cells remaining on the upper side of the membrane were carefully wiped away with cotton swabs. The invaded cells attached to the underside of the membrane were fixed with 4% paraformaldehyde for 15 min and stained with 0.05% crystal violet (C0121, Beyotime, China) for 20 min. Images were captured from three to five randomly chosen microscopic fields, and the invaded cells were counted for quantitative analysis.

### 2.14 Wound healing assay

SKOV3 and OVCAR‐3 cells were seeded into 6‐well plates and grown to 90%–100% confluence. A linear scratch was created across the cell monolayer using a sterile 10 μL pipette tip. Detached cells were removed by washing three times with PBS, after which serum‐free medium was added. Images of the same regions were captured at 0 and 24 h. The wound area was quantified using ImageJ software, and the rate of wound closure was calculated accordingly.

### 2.15 ChIP‐PCR assay

Chromatin immunoprecipitation was performed using a ChIP assay kit (17–295, Millipore, USA). When cells reached approximately 80% confluence, they were cross‐linked with 1% formaldehyde (F8775, Sigma‐Aldrich, USA) for 10 min at room temperature, followed by quenching with 125 mM glycine for 5 min. After PBS washing, cells were lysed with SDS lysis buffer and subjected to sonication using Bioruptor Plus (Diagenode, Belgium), with the number of cycles adjusted to obtain chromatin fragments between 200 and 500 bp. For immunoprecipitation, 2 μg of anti‐HIF1α antibody (NB100‐123, Novus Biologicals, USA) or IgG control antibody (ab172730, Abcam, UK) was added and incubated overnight at 4°C with rotation. After sequential washing, reverse crosslinking, and proteinase K digestion, DNA was recovered and amplified using SYBR green PCR master mix (4367659, Applied Biosystems, USA) on a QuantStudio 5 RT‐PCR system (Thermo Fisher Scientific, USA). PCR conditions were as follows: 95°C for 10 min, followed by 40 cycles of amplification (95°C for 15 s, 60°C for 1 min). To quantify ChIP enrichment, all ChIP samples were normalized to input DNA, and the relative enrichment of HIF1A at the UBR5 promoter region was calculated using the 2^−ΔΔCt^ method. IgG immunoprecipitation was used as the negative control. Each experiment was independently repeated three times, and the mean enrichment fold was calculated for statistical analysis.

### 2.16 Luciferase reporter assay

A reporter plasmid containing the UBR5 promoter fragment (UBR5‐promoter‐WT) was constructed, and the predicted HRE site (ACGTG) was mutated to generate UBR5‐promoter‐MUT. All plasmids were synthesized by Tsingke Biotechnology (Beijing, China). HEK293 T cells (CRL‐3216, ATCC, USA) were seeded in 24‐well plates at a density of 5 × 10^4^ cells per well and co‐transfected with 400 ng of reporter plasmid, 50 ng of the Renilla luciferase internal control plasmid pRL‐TK (E2241, Promega, USA), and 400 ng of oe‐HIF1A or an equivalent amount of empty vector. After 24 h, cells were lysed, and luciferase activities were determined using luciferase assay reagent II and Stop & Glo reagent. Firefly and Renilla luciferase activities were measured using a multifunctional plate reader (SpectraMax iD3, Molecular Devices, USA). The ratio of Firefly to Renilla luminescence was used to evaluate UBR5 promoter activity.

### 2.17 Co‐immunoprecipitation (Co‐IP)

SKOV3 or OVCAR‐3 cells were lysed on ice for 30 min using IP lysis buffer containing protease inhibitors (P0013, Beyotime, China), followed by centrifugation at 12,000 × g for 10 min at 4°C to collect the supernatant. Pre‐washed Protein A/G magnetic beads (Thermo Fisher Scientific, USA) were incubated with either anti‐UBR5 (A13816, ABclonal, China) or anti‐LATS2 (20276‐1‐AP, Proteintech, USA) antibodies at 4°C for 1 h to form antibody‐bead complexes. Equal volumes of cell lysates were then added and incubated at 4°C for an additional 4 h with rotation. The beads were washed four times with IP lysis buffer and boiled in 2 × SDS loading buffer at 95°C for 5 min. The Co‐IP complexes were separated by SDS‐PAGE and subjected to WB analysis to detect interacting proteins such as UBR5 and LATS2. IgG (AC042, ABclonal, China) was used as a negative control.

### 2.18 In vitro ubiquitination assay

Purified LATS2 and UBR5 proteins were expressed in *E*. *coli* BL21(DE3) CodonPlus (RPIL) (Agilent) using the pETDuet‐1 vector (Novagen), and were subsequently purified using cobalt affinity chromatography or S‐tag agarose (Novagen), respectively. The ubiquitination reaction mixture contained 50 mM Tris‐HCl (pH 7.5), 5 mM MgCl_2_, 2 mM ATP (A8270, Solarbio, China), 0.5 μM E1 enzyme UBE1 (AF2710, Beyotime, China), 2 μM E2 enzyme UBE2B (K109529P, Solarbio, China), 1 μg UBR5, 3 μg LATS2, and 10 μg ubiquitin (U5507, Sigma‐Aldrich, USA) in a total volume of 30 μL. The reaction was incubated at 30°C for 1.5 h, and then terminated by adding an equal volume of 2 × SDS loading buffer and heating at 95°C for 5 min. Reaction products were separated by SDS‐PAGE and analyzed by WB to detect LATS2 ubiquitination using an anti‐Ubiquitin antibody (ab7780, Abcam, UK).

### 2.19 In vivo experiment

Female BALB/c nude mice (6–8 weeks old, BALB/cA Slac‐nu, SLAC, China) were housed under specific pathogen‐free conditions (20–24°C, 40%–60% humidity, 12/12 h light/dark cycle) with ad libitum access to food and water. All animal experiments were approved by the Animal Ethics Committee of Suzhou Science & Technology Town Hospital (No. SWAE202618). Surgical procedures were performed under continuous 2% isoflurane inhalation anesthesia, and the absence of pedal reflexes was confirmed prior to applying ophthalmic ointment to protect the cornea.

Mice were placed in a supine position, and the surgical site on the right flank was sterilized with 70% ethanol. A 0.8–1 cm incision was made through the skin and abdominal wall along the lower edge of the right dorsal rib margin to expose the ovarian fat pad. The fat pad was gently pulled out using forceps and placed on a moist sterile gauze. A total of 1 × 10^5^ SKOV3 cells, resuspended in 10 μL PBS containing 2% Matrigel, were slowly injected into the space between the ovarian bursa and ovary using a Hamilton microsyringe (30 G). Upon confirming slight distension of the bursa, the fat pad was returned to the peritoneal cavity and the incision was closed using wound clips.

Seven days after tumor establishment, mice received intraperitoneal injections of GEM (50 mg/kg, 200 μL PBS) twice weekly for 4 weeks. Additionally, mice were administered daily intraperitoneal injections of XMU‐MP‐1 (10 mg/kg, HY‐100526, MedChemExpress) or PBS as control. On day 28, mice were sacrificed, tumor volume was calculated (*V* = 0.5 × length × width^2^), and tumors were weighed for subsequent experiments.

### 2.20 Immunohistochemistry (IHC)

Tissue samples were fixed in 4% paraformaldehyde (P0147 A, Beyotime, China) for 24 h, followed by standard dehydration, clearing, and paraffin embedding procedures. Paraffin sections of 4 μm thickness were prepared. After xylene deparaffinization and rehydration with graded ethanol, antigen retrieval was carried out in EDTA buffer (P0085, Beyotime) using microwave heating. The sections were cooled to room temperature, treated with 3% H_2_O_2_ for 10 min to quench endogenous peroxidase activity, and blocked with 5% goat serum (C0265, Beyotime) for 30 min. Primary antibodies were applied and incubated overnight at 4°C. The primary antibodies used in this study included: HIF‐1α (#48085, Cell Signaling Technology, USA), UBR5 (LS‐B5219‐50, LSBio, USA), LATS2 (ab111054, Abcam, UK), YAP1 (ab205270, Abcam, UK), p‐YAP1 (ab76252, Abcam, UK), and FGFR1 (ab95940, Abcam, UK). After returning to room temperature the next day, sections were incubated with biotinylated secondary antibody (33103ES60, YEASEN, China) for 30 min, followed by incubation with a streptavidin‐HRP complex for 20 min. DAB substrate (P0202, Beyotime) was used for signal development under microscopic observation. After adequate staining, the sections were counterstained with hematoxylin, and dehydrated, cleared, and sealed with neutral resin. Digital slide images were obtained using a Pannoramic Midi scanner (3DHISTECH, Hungary).

### 2.21 Hematoxylin and Eosin (H&E) staining

Tumor tissues were harvested immediately after euthanasia and fixed in 4% paraformaldehyde (P0147 A, Beyotime, China) for 24 h. The samples were subsequently processed through routine dehydration, clearing, and paraffin embedding. Paraffin sections (4 μm) were prepared for staining. The sections were first deparaffinized in xylene and then rehydrated using a graded ethanol series (100%, 95%, 85%, and 75%) followed by distilled water. Hematoxylin staining (C0105 M, Beyotime) was performed for 5 min, after which the sections were rinsed under running water for 1–2 min to allow color development. Eosin staining was then carried out for 15 s. Following staining, the sections were dehydrated with 95% and absolute ethanol, cleared in xylene, and mounted with neutral resin (C1795, Sigma‐Aldrich, USA). Finally, the slides were air‐dried and examined under a light microscope for image acquisition.

### 2.22 Statistical analysis methods

All experiments were repeated at least three times independently, and results are expressed as mean ± standard deviation. Differences between two groups were assessed using an unpaired Student's *t*‐test. For comparisons among three or more groups, one‐way ANOVA was applied, followed by Tukey's HSD post hoc test when significant differences were detected. For datasets that did not meet normality assumptions or showed unequal variance, the Mann–Whitney *U* test or Kruskal–Wallis *H* test was used as appropriate. Statistical analyses were performed with GraphPad Prism 9 and *R* software. A two‐sided *p*‐value <0.05 was considered statistically significant.

## 3 RESULTS

### 3.1 Upregulation of HIF1A in OC and its enrichment in key metabolic and signaling pathways

**FIGURE 1:**
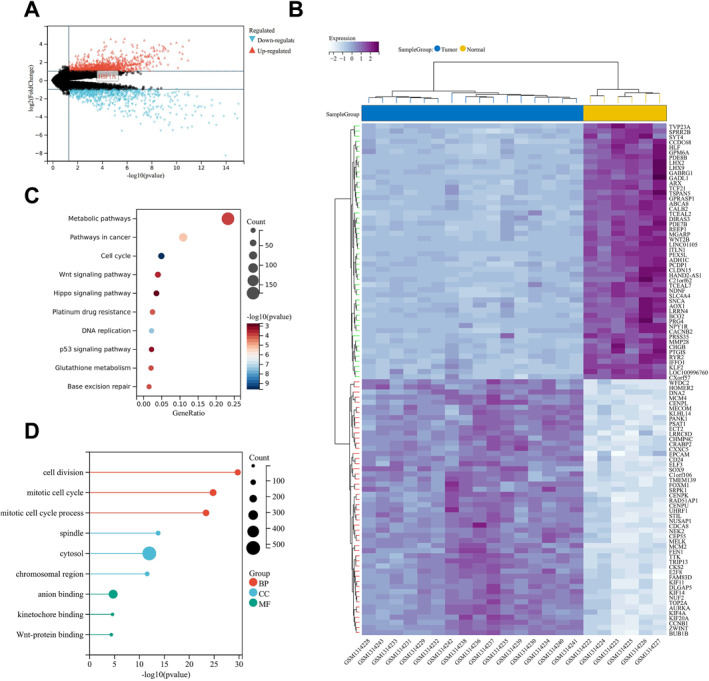
Differential analysis of Wnt and Hippo signaling pathways in ovarian cancer (OC) and normal epithelial tissues. (A) Volcano plot showing differential gene expression analysis between OC and normal epithelial tissues. Red dots represent significantly upregulated genes, blue dots represent significantly downregulated genes, and black dots indicate non‐significant genes; (B) heatmap of the top 50 DEGs; (C, D) functional enrichment analysis of differentially expressed genes, showing results for Kyoto encyclopedia of genes and genomes (C) and gene ontology (D). Tumor: *n* = 16; Normal: *n* = 6.

Differential expression analysis was conducted using the OC‐related transcriptomic dataset GSE54388 obtained from the GEO database. A total of 1844 DEGs were identified, including 1124 upregulated and 720 downregulated genes. Among these, HIF1A, recognized as a critical transcription factor involved in tumor progression and targeted therapy,^28^ was significantly upregulated in OC samples (Figure 1A,B).

Functional enrichment analysis revealed that these DEGs were mainly involved in several key KEGG pathways, including metabolic pathways, Wnt signaling, Hippo signaling, platinum drug resistance, and cytochrome P450‐mediated drug metabolism (Figure 1C). GO analysis further indicated significant enrichment in processes such as the mitotic cell cycle, chromosomal regions, and Wnt protein binding. Notably, the Wnt and Hippo signaling pathways are well known for their roles in regulating cell (Figure 1D). The Wnt and Hippo signaling pathways play critical roles in regulating cell proliferation, migration, and apoptosis, which are closely linked to the aggressiveness and prognosis of OC.^29^, ^30^ Enriching the platinum drug resistance pathway, in particular, highlights potential mechanisms of treatment failure, offering new strategic directions for overcoming chemoresistance.

Overall, the comparative analysis between OC and normal epithelial tissues highlights critical genes and pathways implicated in OC development and progression, with particular emphasis on the elevated expression of HIF1A and its involvement in Wnt and Hippo signaling networks.

### 3.2 Mechanism of HIF1A upregulation in OC and its impact on the Hippo signaling pathway through E3 ubiquitin ligase UBR5

**FIGURE 2:**
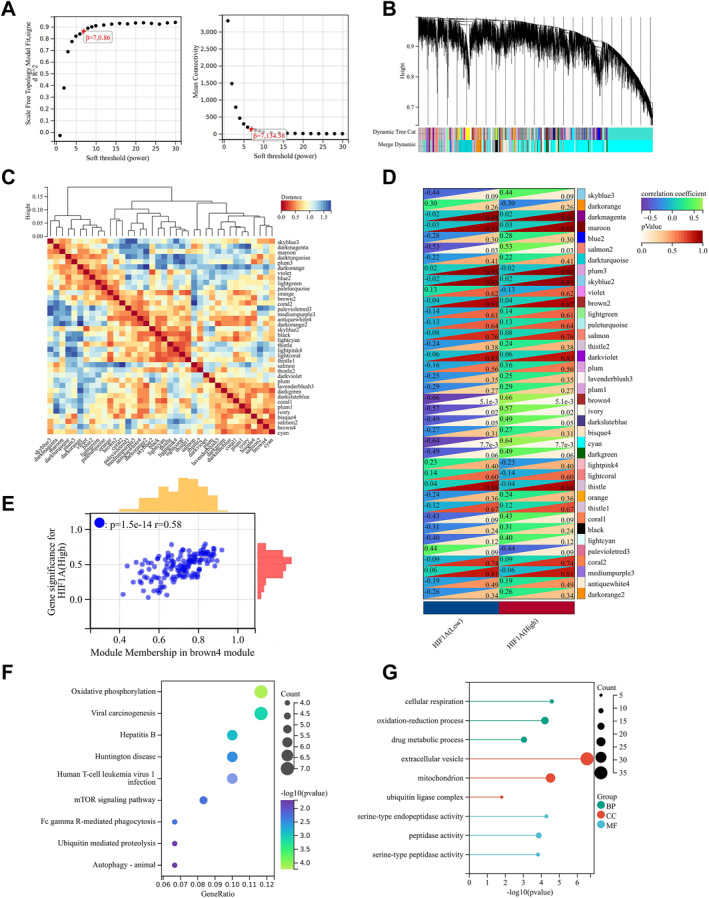
Molecular regulatory mechanisms of hypoxia‐inducible factor 1‐alpha (HIF1A) in ovarian cancer and its biological impact. (A) Scale independence (left) and mean connectivity (right) to determine the weighted *β* value of 7, meeting the scale‐free network criteria; (B) dendrogram of co‐expression network modules; (C) heatmap showing correlations between 38 identified modules; (D) correlation analysis between module expression and HIF1A expression levels (high vs. low); (E) scatter plot of brown4 module genes and HIF1A (High) expression; (F, G) functional enrichment analysis of brown4 module genes, with Kyoto encyclopedia of genes and genomes (F) and gene ontology (G) results. HIF1A (high): *n* = 8; HIF1A (low): *n* = 8.

In this study, we observed a significant increase in the expression of HIF1A in OC epithelial tissues. To delve deeper into the molecular mechanisms downstream of HIF1A, we performed WGCNA analysis on the tumor samples and identified 38 distinct modules (Figure 2A), as illustrated by the clustering dendrogram (Figure 2B). The correlations among these 38 modules are shown in Figure 2C.

To identify gene modules associated with HIF1A, samples were stratified into high‐ and low‐HIF1A expression groups. Among the detected modules, the brown4 module displayed the strongest positive association with high HIF1A expression (cor = 0.66, *p* = 5.1 × 10^−3^). Its module eigengene was also positively correlated with gene significance (cor = 0.58, *p* = 1.5 × 10^−14^) (Figure 2D,E).

We identified a module of 149 genes significantly associated with HIF1A expression. Enrichment analysis of these genes revealed significant involvement in oxidative phosphorylation, mTOR signaling pathway, and ubiquitin‐mediated proteolysis (Figure 2F). GO enrichment results indicated significant associations with oxidation‐reduction processes, drug metabolic processes, mitochondria, ubiquitin ligase complexes, and peptidase activity (Figure 2G). Notably, ubiquitination is an important post‐translational modification involved in cancer initiation and progression through regulation of transcriptional, translational, and post‐translational events.^31^, ^32^ Therefore, this study further focused on the potential role of ubiquitination‐related pathways among HIF1A‐associated genes.

**FIGURE 3:**
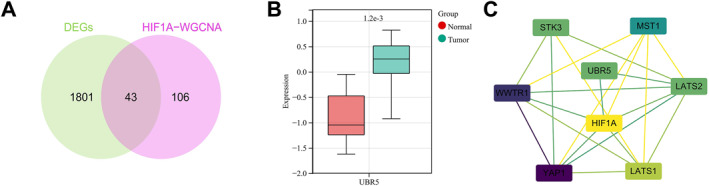
Molecular mechanism of hypoxia‐inducible factor 1‐alpha (HIF1A) regulation via ubiquitination network in ovarian cancer. (A) Venn diagram showing the overlap between brown 4 module genes and DEGs; (B) box plot illustrating UBR5 expression levels in the transcriptome dataset (Tumor: *n* = 16; Normal: *n* = 6); (C) PPI network of HIF1A, UBR5, and critical factors from the Hippo signaling pathway.

The genes from the brown4 module were intersected with the DEGs, yielding 43 overlapping genes (Figure 3A). Among them, UBR5—a gene encoding an E3 ubiquitin ligase of the HECT family—is frequently amplified in breast, ovarian, and prostate cancers.^33^ Compared with the normal group, UBR5 was significantly upregulated in tumor samples (Figure 3B). To evaluate the diagnostic potential of HIF1A and UBR5 in distinguishing tumor from normal tissues, ROC curve analyses were performed. The AUC values for HIF1A and UBR5 were 0.833 and 0.927, respectively, indicating strong discriminative ability (Supporting Information S1; Figure S1A). Subsequently, we analyzed the expression levels of HIF1A and UBR5 in the TCGA OC dataset. HIF1A expression was significantly higher in tumor tissues than in normal tissues, and UBR5 expression was markedly elevated in the HIF1A high‐expression group compared with the low‐expression group (Supporting Information S1; Figure S1B). Pearson correlation analysis further showed a positive association between the HIF1A and UBR5 expressions (*r* = 0.526, *p* < 0.001) (Supporting Information S1; Figure S1C), supporting their potential involvement in OC progression.

Moreover, enrichment analysis indicated that the DEGs were significantly associated with the Hippo signaling pathway. Aberrant Hippo pathway activity was reported to promote tumorigenesis in model systems and is frequently observed in multiple human malignancies, including OC.^30^, ^34^ Various E3 ubiquitin ligases have been shown to inactivate the Hippo signaling pathway.^21^, ^22^

Lastly, we used the STRING database to conduct a PPI analysis of HIF1A, UBR5, and several key factors of the Hippo signaling pathway, revealing interactions among them (Figure 3C).

Through WGCNA and enrichment analyses, this study has revealed the molecular regulatory network of HIF1A in OC, particularly highlighting the role of the E3 ubiquitin ligase UBR5 in the Hippo signaling pathway. These findings not only elucidate the molecular mechanisms associated with HIF1A but also underscore the critical role of ubiquitination in OC, providing potential therapeutic strategies targeting these molecular pathways.

### 3.3 GEM suppresses the expression of HIF1A and UBR5

**FIGURE 4:**
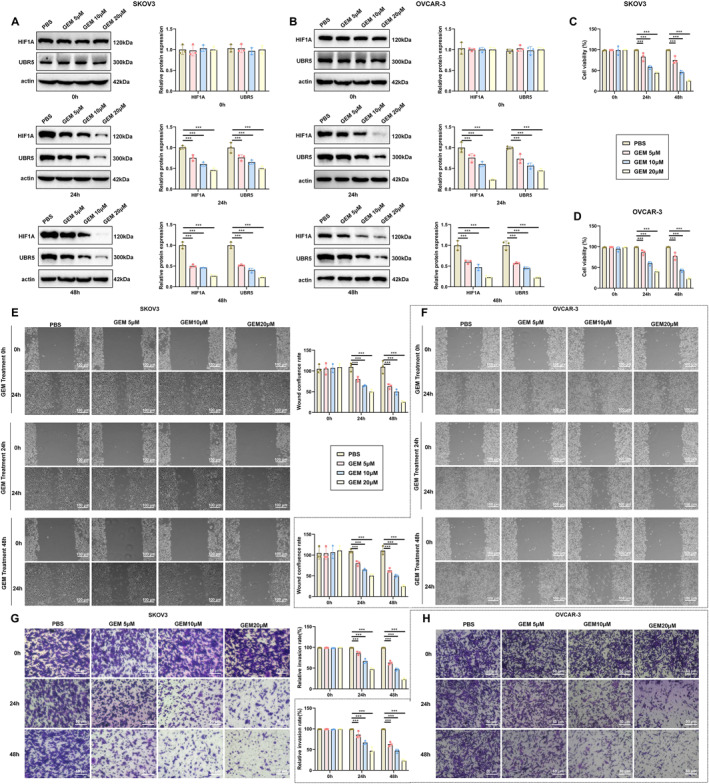
Effects of GEM on hypoxia‐inducible factor 1‐alpha (HIF1A), UBR5, and ovarian cancer Cells. (A) Western blot (WB) analysis of HIF1A and UBR5 expression in SKOV3 cells under hypoxic conditions treated with different concentrations of GEM; (B) WB analysis of HIF1A and UBR5 expression in OVCAR‐3 cells under hypoxic conditions treated with different concentrations of GEM; (C) CCK‐8 assay of cell viability in SKOV3 cells treated with varying concentrations of GEM at 0, 24, and 48 h; (D) CCK‐8 assay of cell viability in OVCAR‐3 cells treated with varying concentrations of GEM at 0, 24, and 48 h; (E) wound healing assay assessing the migration rate of SKOV3 cells treated with different concentrations of GEM at 0 h, 24 h, and 48 h (scale bar: 100 μm); (F) wound healing assay assessing the migration rate of OVCAR‐3 cells treated with different concentrations of GEM at 0, 24, and 48 h (scale bar: 100 μm); (G) transwell invasion assay evaluating the invasive capacity of SKOV3 cells treated with different concentrations of GEM at 0, 24, and 48 h (scale bar: 50 μm); (H) transwell invasion assay evaluating the invasive capacity of OVCAR‐3 cells treated with different concentrations of GEM at 0, 24, and 48 h (scale bar: 50 μm). Data are presented as mean ± SD; all cellular experiments were performed in triplicate. ***p < 0.001, analyzed using ANOVA followed by Tukey's multiple comparison test.

GEM is considered a safe and effective chemotherapeutic agent for OC treatment,^35^, ^36^ yet the specific molecular mechanisms by which GEM inhibits OC remain unclear. Bioinformatics analysis has shown that HIF1α and UBR5 are strongly associated with OC and are highly expressed within the disease context, suggesting that GEM's effectiveness may be mediated through the suppression of HIF1α and UBR5, thereby inhibiting tumor growth and invasion. To test this hypothesis, we first evaluated the effects of GEM on OC cells using SKOV3 and OVCAR‐3 cell lines as in vitro models. To stably detect the expression of HIF1A protein, all cells were incubated under hypoxic conditions (1% O_2_) in a hypoxia chamber prior to and during the treatment period. WB results indicated no significant change in the protein levels of HIF1α and UBR5 in the SKOV3 cells at 0 h across all concentrations compared to the PBS group. However, after 24 h of treatment, a concentration‐dependent decrease in the protein levels of HIF1α and UBR5 was observed, starting with the 5 μM GEM group, with further reductions as the concentration increased. This pattern persisted after 48 h of treatment, with the protein levels continuing to decline in a concentration‐dependent manner (Figure 4A). A similar outcome was observed in OVCAR‐3 cells under the same treatment conditions (Figure 4B).

CCK‐8 results showed that GEM had no obvious effect on SKOV3 cell viability at 0 h at any tested concentration. After 24 h of treatment, however, cell viability was reduced in the 5 μM GEM group compared with the PBS group, and this inhibitory effect became stronger as the GEM concentration increased. A similar concentration‐dependent reduction was also observed at 48 h (Figure 4C). Similar results were obtained with OVCAR‐3 cells under the same conditions (Figure 4D). The wound healing assay assessed the impact of GEM on the migration rate of SKOV3 and OVCAR‐3 cells. No significant changes in cell motility were observed immediately after treatment at 0 h. However, after 24 h, the migration rate decreased in the 5 μM GEM group compared to the PBS group, with further reductions as the concentration increased. This concentration‐dependent decrease continued at 48 h (Figure 4E). A similar pattern was observed in OVCAR‐3 cells (Figure 4F). The transwell assay was used to evaluate the invasive potential of SKOV3 and OVCAR‐3 cells following treatment with GEM. At 0 h, there was no significant impact on cell invasiveness at any concentration. After 24 h, invasiveness decreased in the 5 μM GEM group compared to PBS, with further reductions as the concentration increased, demonstrating a concentration‐dependent decrease. This trend was also evident at 48 h (Figure 4G). OVCAR‐3 cells showed comparable results under identical conditions (Figure 4H).

We conducted rescue experiments to verify the critical roles of HIF1A and UBR5 in the efficacy of GEM. In SKOV3 and OVCAR‐3 cells, we overexpressed HIF1A and UBR5 individually to determine their regulatory relationship and how GEM affects these factors to subsequently inhibit the progression of OC. To stably detect HIF1A protein expression, all cells were cultured in a hypoxic incubator (1% O_2_) before and during treatment. The results indicated that the optimal treatment regimen involved administering 20 μM of GEM for 48 h; thus, subsequent experiments utilized this system to treat the cells with GEM.

WB analysis showed that compared to the oe‐NC group, the expression of HIF1A and UBR5 proteins increased in the oe‐HIF1A groups of SKOV3 and OVCAR‐3 cells. In contrast, only UBR5 expression increased in the oe‐UBR5 groups, whereas HIF1A levels remained unchanged (Supporting Information S1; Figure S2A,B). This suggests a regulatory hierarchy between HIF1A and UBR5, prompting further investigation into whether GEM impacts OC cell phenotypes through these proteins. CCK‐8 analysis showed that treatment with 20 μM GEM for 48 h significantly reduced the viability of both SKOV3 and OVCAR‐3 cells compared with the PBS group. Notably, cell viability was partially restored in the GEM + oe‐HIF1A and GEM + oe‐UBR5 groups relative to the GEM + oe‐NC group (Supporting Information S1; Figure S2C,D). Consistently, wound healing assays demonstrated that GEM treatment markedly suppressed cell migration compared with PBS controls, whereas reintroduction of HIF1A or UBR5 attenuated this inhibitory effect (Supporting Information S1; Figure S2E,F). Similarly, transwell assays revealed reduced invasive capacity in GEM‐treated cells, whereas overexpression of HIF1A or UBR5 increased invasion compared with the GEM + oe‐NC group (Supporting Information S1; Figure S2G,H). Collectively, these findings suggest that GEM suppresses HIF1A and UBR5 expression in OC cells, thereby inhibiting cell proliferation, migration, and invasion.

### 3.4 GEM suppresses OC cell proliferation and invasion by inhibiting HIF1A expression and downregulating UBR5

**FIGURE 5:**
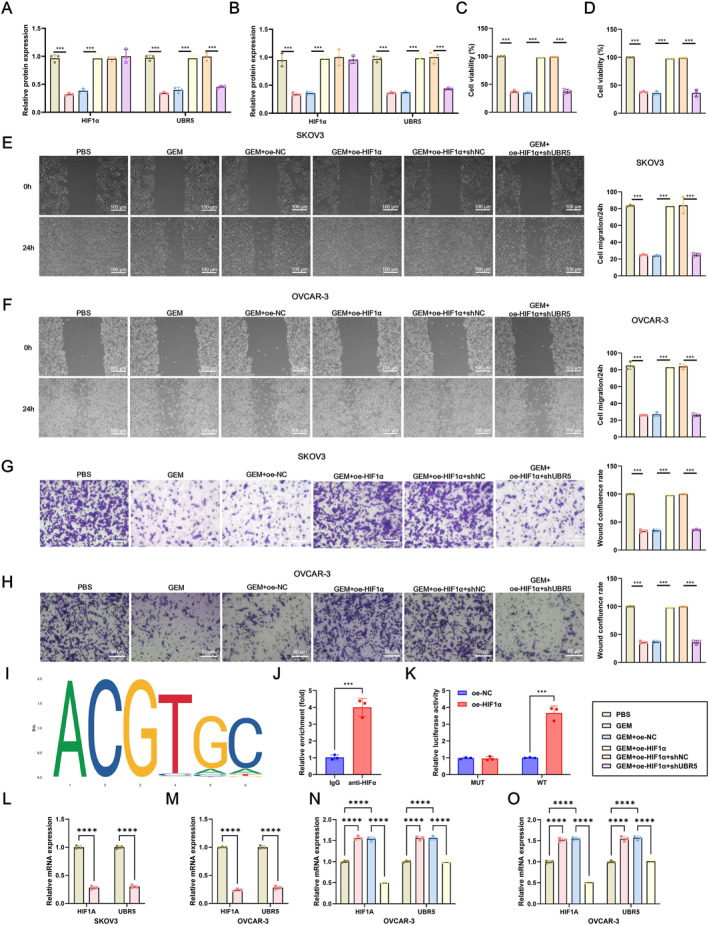
Effects of GEM on ovarian cancer proliferation and invasion via HIF1A‐mediated downregulation of UBR5. (A) Western blot (WB) analysis of hypoxia‐inducible factor 1‐alpha (HIF1A) and UBR5 expression levels in SKOV3 cells treated with 20 μM GEM for 48 h; (B) WB analysis of HIF1A and UBR5 expression levels in OVCAR‐3 cells treated with 20 μM GEM for 48 h; (C) CCK8 assay showing cell viability of SKOV3 cells treated with 20 μM GEM for 48 h; (D) CCK8 assay showing cell viability of OVCAR‐3 cells treated with 20 μM GEM for 48 h; (E) wound healing assay assessing the migration rate of SKOV3 cells treated with 20 μM GEM for 48 h (Scale bar: 100 μm); (F) wound healing assay assessing the migration rate of OVCAR‐3 cells treated with 20 μM GEM for 48 h (Scale bar: 100 μm); (G) transwell invasion assay showing the invasive capacity of SKOV3 cells treated with 20 μM GEM for 48 h (Scale bar: 50 μm); (H) transwell invasion assay showing the invasive capacity of OVCAR‐3 cells treated with 20 μM GEM for 48 h (Scale bar: 50 μm); (I) predicted transcription factor binding sites in the UBR5 promoter region based on JASPAR database analysis; (J) ChIP‐PCR analysis showing HIF1A enrichment at the UBR5 promoter region; (K) dual‐luciferase reporter assay evaluating the regulatory effect of HIF1A on UBR5 promoter activity; (L, M) RT‐qPCR analysis of HIF1A and UBR5 mRNA expression levels after shHIF1A knockdown in SKOV3 and OVCAR‐3 cells; (N, O) RT‐qPCR analysis of UBR5 mRNA expression levels in SKOV3 and OVCAR‐3 cells under normoxia, hypoxia, hypoxia + shNC, and hypoxia + shHIF1A conditions. Data are presented as mean ± SD; all cell experiments were performed in triplicate. ***p < 0.001, analyzed by ANOVA followed by Tukey's multiple comparison test.

Bioinformatics analysis has shown that HIF1A is significantly upregulated in OC and closely correlated with UBR5. Based on these findings, it is hypothesized that GEM impacts OC phenotypes by modulating HIF1A expression, which regulates UBR5. To verify this hypothesis, SKOV3 and OVCAR‐3 cells were maintained under hypoxic conditions (1% O_2_) before and during treatment, and protein expression was examined by WB. Compared with the PBS group, GEM treatment reduced HIF1A and UBR5 protein levels in both cell lines. In contrast, HIF1A overexpression increased the expression of both HIF1A and UBR5 compared with the GEM + oe‐NC group. Further comparison showed that UBR5 knockdown in the GEM + oe‐HIF1α + shUBR5 group decreased UBR5 protein expression without altering HIF1A levels relative to the GEM + oe‐HIF1α + shNC group (Figure 5A,B). CCK‐8 assays showed that GEM significantly inhibited cell viability compared with PBS in both SKOV3 and OVCAR‐3 cells. This inhibitory effect was weakened by HIF1A overexpression, as indicated by increased viability in the GEM + oe‐HIF1α group compared with the GEM + oe‐NC group. However, UBR5 knockdown reversed this effect, leading to reduced viability in the GEM + oe‐HIF1α + shUBR5 group compared with the GEM + oe‐HIF1α + shNC group (Figure 5C,D).

Wound healing results showed that GEM treatment significantly reduced the migratory capacity of SKOV3 and OVCAR‐3 cells compared with PBS treatment. Conversely, HIF1α overexpression partially restored cell migration under GEM treatment, as reflected by the higher migration rate in the GEM + oe‐HIF1α group than in the GEM + oe‐NC group. However, this effect was weakened after UBR5 knockdown, with reduced migration observed in the GEM + oe‐HIF1α + shUBR5 group compared with the GEM + oe‐HIF1α + shNC group in both cell lines (Figure 5E,F). Similarly, transwell assays indicated that GEM suppressed the invasive ability of SKOV3 and OVCAR‐3 cells relative to the PBS group. HIF1α overexpression enhanced invasion compared with the GEM + oe‐NC group, whereas UBR5 silencing reversed this increase, leading to lower invasion in the GEM + oe‐HIF1α + shUBR5 group than in the GEM + oe‐HIF1α + shNC group (Figure 5G,H).

To investigate whether HIF1A directly regulates UBR5 at the transcriptional level, we first analyzed the UBR5 promoter region using the JASPAR database and identified a typical HRE binding site: CAACGTGTAG (Figure 5I). Subsequently, ChIP‐PCR was performed to validate the enrichment of HIF1A at the UBR5 promoter. Compared with the IgG control, HIF1A antibody was significantly enriched at the HRE region of the UBR5 promoter (enrichment fold = 4.82 ± 0.53, *p* < 0.001), confirming the specific binding of HIF1A to this region (Figure 5J). To validate the transcriptional regulatory role of this binding site, a dual‐luciferase reporter system was established using a wild‐type UBR5 promoter construct (UBR5‐promoter‐WT) and a corresponding mutant in which the HRE motif was disrupted (UBR5‐promoter‐MUT). The assay results demonstrated that overexpression of HIF1A markedly increased the luciferase activity of the wild‐type promoter, whereas this enhancement was lost in the mutant construct lacking a functional HRE site (Figure 5K).

In addition, HIF1A knockdown experiments were performed in SKOV3 and OVCAR‐3 cells. RT‐qPCR showed that HIF1A mRNA expression was significantly decreased in the shHIF1A group compared with the shNC group, confirming knockdown efficiency; UBR5 mRNA expression was also significantly decreased, indicating that HIF1A knockdown downregulated UBR5 transcription (Figure 5L,M). To determine whether UBR5 upregulation under physiologically relevant hypoxic conditions depends on HIF1A, cells were cultured under hypoxia (1% O_2_) for 48 h. RT‐qPCR showed that HIF1A and UBR5 mRNA levels were significantly increased in the hypoxia group compared with the normoxia group; however, compared with the hypoxia + shNC group, hypoxia‐induced UBR5 mRNA upregulation was significantly suppressed in the hypoxia + shHIF1A group and approached the normoxia level (Figure 5N,O). These results indicate that hypoxia‐induced transcriptional upregulation of UBR5 depends on HIF1A expression. Together, these findings indicate that HIF1A directly binds to and activates the UBR5 promoter, thereby promoting its transcription. GEM inhibits HIF1A expression, resulting in downregulation of UBR5 and ultimately suppressing the proliferation and invasion of OC cells.

### 3.5 GEM regulates LATS2 ubiquitination via the HIF1A/UBR5 axis

This study further investigates how GEM modulates the OC phenotype by regulating the HIF1a/UBR5 axis, which affects the Hippo signaling pathway. Bioinformatics analyses revealed that differential genes in OC are enriched in the Hippo pathway, a crucial cell proliferation and differentiation regulator. Abnormalities in the Hippo pathway are common in various cancers. In the molecular regulatory network, UBR5 interacts with the key Hippo protein LATS2, which promotes the phosphorylation of YAP1. Phosphorylated YAP1 cannot enter the nucleus to activate transcription factors for growth factors, thereby maintaining the activation of the Hippo pathway and inhibiting tumor growth.^37^ The ubiquitination capability of UBR5 may lead to LATS2 instability, resulting in dephosphorylation of YAP1 and its nuclear translocation to promote the expression of pro‐tumorigenic factors.^37^ Growth factor FGFR1, known to be involved in malignant transformation and tumor proliferation in cancers,^38^ has been reported to increase with YAP1 upregulating FGFR1 expression, promoting proliferation in glioblastoma.^39^ Therefore, this study also focuses on exploring the relationship between GEM, the Hippo pathway, and FGFR1 and their collective impact on the progression of OC. To further verify this hypothesis, we first evaluated the clinical relevance of YAP1 and FGFR1 in clinical samples using the TCGA OC dataset. Group comparison and correlation analyses showed that YAP1 and FGFR1 expression levels were significantly higher in the HIF1A‐high group than in the HIF1A‐low group (Figure S3A‐B), suggesting that their expression may be closely associated with HIF1A levels. Pearson correlation analysis further showed that HIF1A was significantly positively correlated with YAP1 expression (*r* = 0.354, *p* < 0.001) and FGFR1 expression (*r* = 0.188, *p* < 0.001) (Figure S3C‐D). These results indicate that the expression of YAP1 and FGFR1 is positively associated with HIF1A in OC tissues, providing clinical bioinformatics support for further investigation of the HIF1A/UBR5‐Hippo‐YAP1/FGFR1 regulatory axis.

**FIGURE 6:**
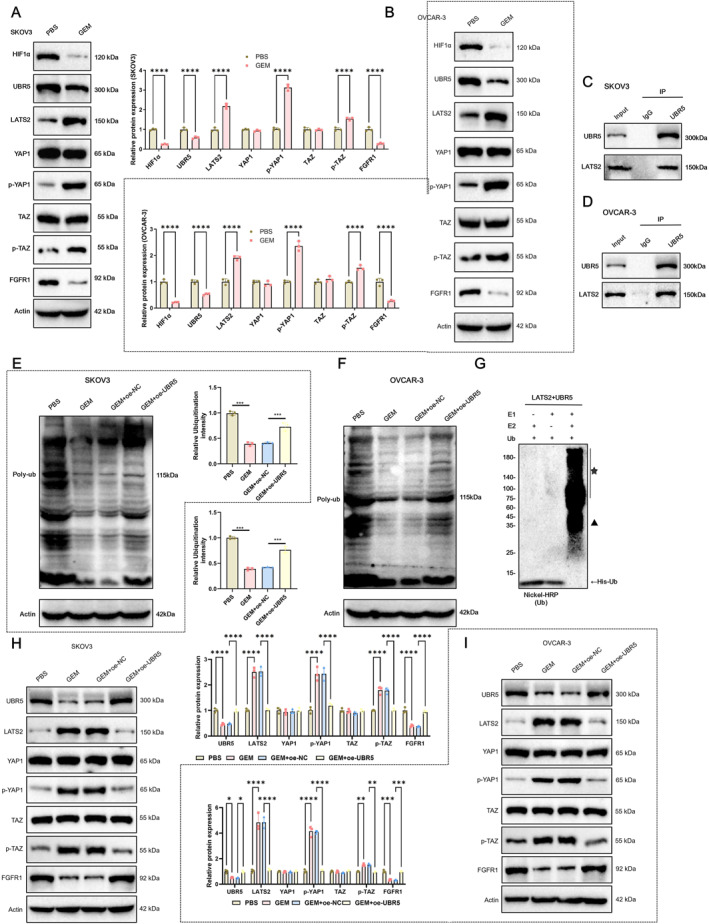
GEM regulates LATS2 ubiquitination and YAP/TAZ phosphorylation via the hypoxia‐inducible factor 1‐alpha/UBR5 axis. (A) Western blot (WB) analysis of Hippo pathway‐related proteins in SKOV3 cells under hypoxic conditions; (B) WB analysis of Hippo pathway‐related proteins in OVCAR‐3 cells under hypoxic conditions; (C) co‐IP analysis showing the interaction between LATS2 and UBR5 in SKOV3 cells; (D) Co‐IP analysis showing the interaction between LATS2 and UBR5 in OVCAR‐3 cells; (E) ubiquitination assay measuring LATS2 ubiquitination levels in SKOV3 cells treated with 20 μM GEM for 48 h; (F) ubiquitination assay measuring LATS2 ubiquitination levels in OVCAR‐3 cells treated with 20 μM GEM for 48 h; (G) WB analysis of in vitro ubiquitination to evaluate UBR5‐mediated LATS2 ubiquitination; (H) WB analysis of Hippo pathway‐related proteins in SKOV3 cells treated with 20 μM GEM for 48 h; (I) WB analysis of Hippo pathway‐related proteins in OVCAR‐3 cells treated with 20 μM GEM for 48 h. Results are presented as mean ± standard deviation. All experiments were repeated three times. ***p < 0.001, using ANOVA and Tukey's multiple comparison test.

To assess HIF1A protein expression, all cells were cultured in a hypoxic incubator (1% O_2_) before and during treatment. WB analysis showed that, in SKOV3 and OVCAR‐3 cells, GEM did not change the expression of the key Hippo pathway protein YAP1 compared with the PBS group, downregulated the cancer‐related growth factor FGFR1, and upregulated LATS2 and p‐YAP1. The protein expression of TAZ and its phosphorylated form p‐TAZ was also examined. Compared with the PBS group, GEM treatment did not markedly change total TAZ protein levels, but significantly increased p‐TAZ protein levels, with a phosphorylation trend consistent with p‐YAP1 (Figure 6A,B). These results indicate that GEM can affect the Hippo pathway and suppress FGFR1 expression, and that this regulatory effect involves phosphorylation activation of both YAP and TAZ. Co‐IP further revealed interactions between LATS2 and UBR5 in these cells (Figure 6C,D). To investigate whether GEM modulates LATS2 ubiquitination through UBR5 regulation, ubiquitination assays were performed. The results showed that GEM treatment reduced LATS2 ubiquitination levels in SKOV3 and OVCAR‐3 cells compared to the PBS group. In contrast, LATS2 ubiquitination levels increased in the GEM + oe‐UBR5 group compared to the GEM + oe‐NC group (Figure 6E,F). Further in vitro ubiquitination assays were conducted by expressing and purifying UBR5 and LATS2, followed by the construction of a reaction system containing E1 and E2 enzymes, ubiquitin, and ATP. After incubation, the ubiquitination level of LATS2 was assessed. The results confirmed that UBR5 functions as an E3 ubiquitin ligase for LATS2 (Figure 6G). Further WB analysis showed that, compared with PBS, GEM did not change total YAP1 or TAZ protein levels, significantly increased p‐YAP1 and p‐TAZ protein levels, decreased UBR5 and FGFR1 protein levels, and increased LATS2 protein levels. Compared with GEM + oe‐NC, GEM + oe‐UBR5 did not change total YAP1 or TAZ protein levels, significantly decreased p‐YAP1 and p‐TAZ protein levels, increased UBR5 and FGFR1 protein levels, and decreased LATS2 protein levels (Figure 6H,I). The results indicated that GEM inhibited the ubiquitination of LATS2 by reducing UBR5 expression, thereby preventing LATS2 degradation and ultimately affecting downstream proteins in the Hippo pathway.

### 3.6 GEM modulates the Hippo pathway by regulating LATS2 ubiquitination via the HIF1A/UBR5 axis

**FIGURE 7:**
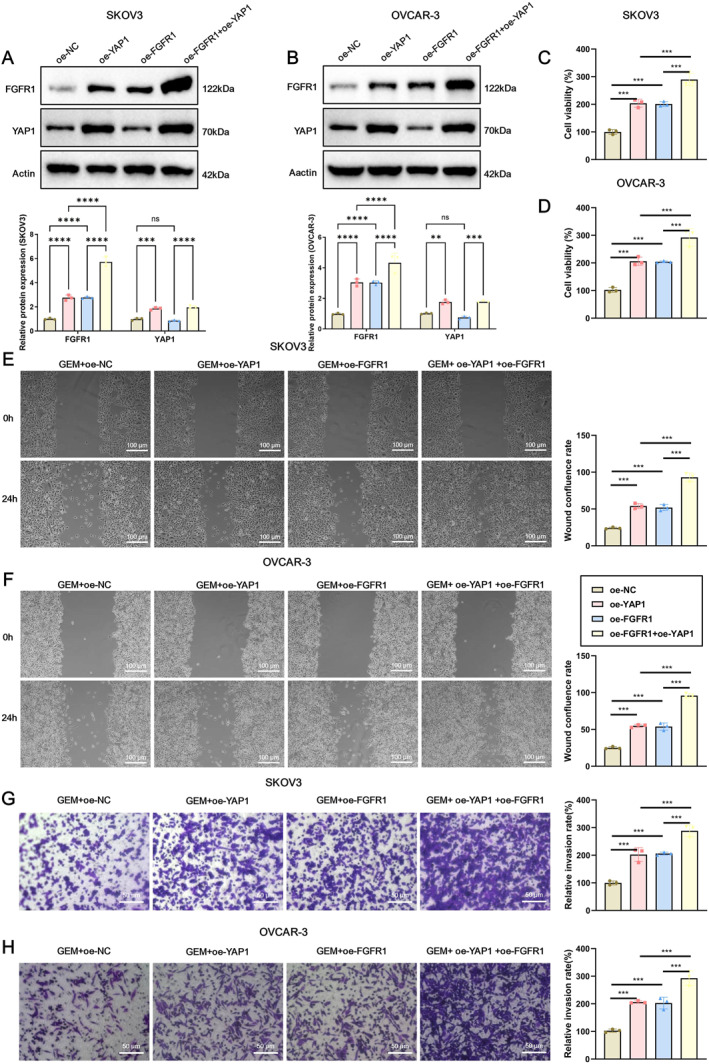
Investigation of GEM's regulation of the Hippo pathway in ovarian cancer inhibition. (A) Western blot (WB) analysis of YAP1 and FGFR1 expression levels in SKOV3 cells treated with 20 μM GEM for 48 h; (B) WB analysis of YAP1 and FGFR1 expression levels in OVCAR‐3 cells treated with 20 μM GEM for 48 h; (C) CCK8 assay measuring cell viability in SKOV3 cells treated with 20 μM GEM for 48 h; (D) CCK8 assay measuring cell viability in OVCAR‐3 cells treated with 20 μM GEM for 48 h; (E) wound healing assay showing migration rates of SKOV3 cells treated with 20 μM GEM for 48 h (scale bar: 100 μm); (F) wound healing assay showing migration rates of OVCAR‐3 cells treated with 20 μM GEM for 48 h (scale bar: 100 μm); (G) transwell assay showing invasion ability of SKOV3 cells treated with 20 μM GEM for 48 h (scale bar: 50 μm); (H) transwell assay showing invasion ability of OVCAR‐3 cells treated with 20 μM GEM for 48 h (scale bar: 50 μm). Results are presented as mean ± standard deviation. Cell experiments were repeated 3 times; ***p < 0.001, using ANOVA and Tukey's multiple comparison test.

To investigate whether GEM inhibits OC progression by regulating the Hippo pathway, we constructed cell lines in SKOV3 and OVCAR‐3 that individually or co‐express the Hippo pathway core transcription factor YAP1 and the key tumor growth factor FGFR1. WB analysis showed that compared to the oe‐NC group, YAP1 and FGFR1 protein expression increased in the oe‐YAP1 group. In the oe‐FGFR1 group, FGFR1 expression increased, whereas YAP1 expression remained unchanged. Compared to the oe‐YAP1 group, YAP1 expression remained unchanged in the oe‐FGFR1 + oe‐YAP1 group, but FGFR1 expression increased. Additionally, compared to the oe‐FGFR1 group, both YAP1 and FGFR1 expression increased in the oe‐FGFR1 + oe‐YAP1 group, indicating that YAP1 is an upstream regulator of FGFR1 (Figure 7A,B). CCK‐8 analysis showed that YAP1 overexpression increased cell viability under GEM treatment compared with the GEM + oe‐NC group in both SKOV3 and OVCAR‐3 cells. A similar increase was observed after FGFR1 overexpression. Notably, simultaneous overexpression of YAP1 and FGFR1 further enhanced cell viability compared with either YAP1 or FGFR1 overexpression alone (Figure 7C,D).

Wound healing assays showed that, under GEM treatment, overexpression of YAP1 increased the migratory ability of both SKOV3 and OVCAR‐3 cells compared with the oe‐NC group. FGFR1 overexpression produced a similar enhancement in cell migration. Moreover, co‐overexpression of YAP1 and FGFR1 further promoted migration relative to either single‐gene overexpression group (Figure 7E,F). Transwell assays further indicated that YAP1 overexpression enhanced cell invasion compared with GEM + oe‐NC treatment in both cell lines. A comparable increase was observed in the GEM + oe‐FGFR1 group. Notably, simultaneous overexpression of YAP1 and FGFR1 resulted in a stronger invasive phenotype than overexpression of either YAP1 or FGFR1 alone (Figure 7G,H).

These results suggest that GEM regulates the Hippo pathway, inhibiting FGFR1 expression and ultimately alleviating the progression of OC.

### 3.7 GEM inhibits the HIF1A/UBR5 axis to modulate the Hippo Pathway and suppress OC progression in vivo

**FIGURE 8:**
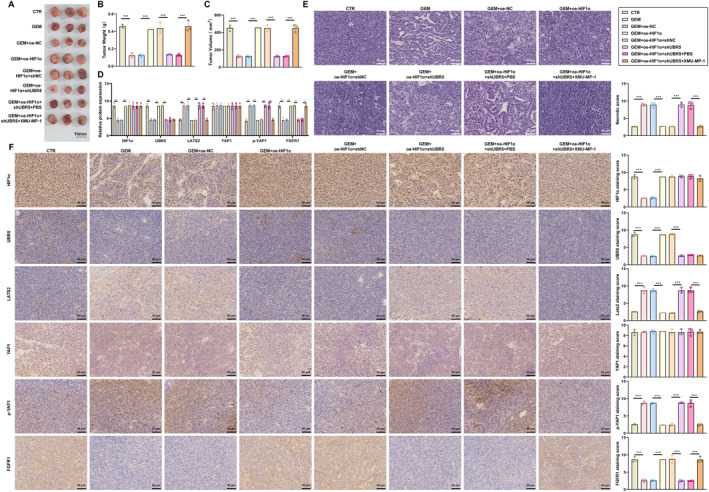
Investigation of GEM's inhibition of the hypoxia‐inducible factor 1‐alpha (HIF1A)/UBR5 axis regulating the Hippo pathway in ovarian cancer in vivo. (A) Tumor tissue morphology of mice; (B) tumor mass statistics for mice; (C) tumor size statistics for mice; (D) Western blot analysis of HIF1A, UBR5, Hippo pathway‐related proteins, and FGFR1 expression in tumor tissue; (E) H&E staining analysis of tumor necrosis (Scale bar: 50 μm); (F) IHC analysis of HIF1A, UBR5, Hippo pathway‐related proteins, and FGFR1 expression in tumor tissue (Scale bar: 50 μm). Data are presented as mean ± standard deviation (*n* = 6); ***p < 0.001, using ANOVA and Tukey's multiple comparison test.

Finally, we validated the therapeutic effect of GEM on OC in vivo using an orthotopic OC mouse model. The mice were injected with OC cells overexpressing HIF1α or with UBR5 knockdown and treated with the Hippo inhibitor XMU‐MP‐1 ^37^ to assess GEM's anti‐tumor mechanism in vivo. Tumor evaluation showed that GEM treatment reduced tumor volume and weight compared with the CTR group. However, HIF1α overexpression weakened this antitumor effect, resulting in larger tumors than those in the CTR + GEM + oe‐NC group. In contrast, UBR5 knockdown reduced tumor growth in the CTR + GEM + oe‐HIF1α + shUBR5 group compared with the CTR + GEM + oe‐HIF1α + shNC group. Moreover, administration of XMU‐MP‐1 increased tumor size and weight compared with the PBS treatment in the CTR + GEM + oe‐HIF1α + shUBR5 background (Figure 8A–C).

WB analysis showed that, compared to the CTR group, the CTR + GEM group exhibited no change in YAP1 expression, whereas HIF1A, UBR5, and FGFR1 protein levels decreased, and LATS2 and p‐YAP1 levels increased. Compared to the CTR + GEM + oe‐NC group, the CTR + GEM + oe‐HIF1α group showed no change in YAP1 expression but an increase in HIF1A, UBR5, and FGFR1 protein levels, with a decrease in LATS2 and p‐YAP1 levels. Furthermore, compared to the CTR + GEM + oe‐HIF1α + shNC group, the CTR + GEM + oe‐HIF1α + shUBR5 group displayed unchanged levels of HIF1A and YAP1, but a decrease in UBR5 and FGFR1 protein levels, with an increase in LATS2 and p‐YAP1. Additionally, compared to the CTR + GEM + oe‐HIF1α + shUBR5 + PBS group, the CTR + GEM + oe‐HIF1α + shUBR5 + XMU‐MP‐1 group exhibited no change in HIF1A, UBR5, and YAP1 expression, whereas FGFR1 protein levels increased, and LATS2 and p‐YAP1 levels decreased (Figure 8D). H&E staining of tumor necrosis revealed that, compared to the CTR group, the necrosis index of the CTR + GEM group increased. Compared to the CTR + GEM + oe‐NC group, the necrosis index decreased in the CTR + GEM + oe‐HIF1α group. In contrast, the necrosis index increased in the CTR + GEM + oe‐HIF1α + shUBR5 group compared to the CTR + GEM + oe‐HIF1α + shNC group. Finally, compared to the CTR + GEM + oe‐HIF1α + shUBR5 + PBS group, the necrosis index decreased in the CTR + GEM + oe‐HIF1α + shUBR5 + XMU‐MP‐1 group (Figure 8E). The protein expression pattern observed by IHC was entirely consistent with the WB results (Figure 8F). These findings suggest that GEM suppressed OC progression in vivo by inhibiting the HIF1A/UBR5 axis and subsequently modulating the Hippo signaling pathway.

## 4 DISCUSSION

GEM, a nucleoside analog, has been widely used in treating various cancers,^40^ particularly showing potential in inhibiting OC cell growth.^36^, ^41^ By mimicking intracellular nucleotides, GEM disrupts DNA replication, inhibiting cancer cell proliferation.^24^, ^40^ Understanding how GEM exerts these effects through molecular mechanisms could lead to more effective therapeutic strategies for OC.

In this study, we systematically investigated the inhibitory effects of GEM on OC cells and its potential molecular mechanisms. Analysis of OC‐related transcriptomic data from the GEO database revealed significantly elevated expression levels of HIF1A and UBR5 in OC tissues. HIF1A, a key transcription factor, is closely linked to cancer progression, whereas UBR5, an E3 ubiquitin ligase, plays a crucial role in protein degradation and cellular signaling. Our WGCNA analysis identified gene modules closely associated with HIF1A, elucidating how GEM regulates the HIF1A/UBR5 axis to modulate the Hippo signaling pathway, thereby limiting tumor growth.

In vitro, SKOV3 and OVCAR‐3 cells were exposed to different concentrations of GEM, which markedly downregulated HIF1A and UBR5 expression in both a dose‐ and time‐dependent manner. Further analyses confirmed that HIF1A, acting as a transcription factor, directly binds to the UBR5 promoter and promotes its transcription. This regulatory relationship was supported by JASPAR prediction, ChIP‐PCR validation, and dual‐luciferase reporter assays. These findings indicate that GEM suppresses HIF1A expression, thereby reducing UBR5 transcription. Decreased UBR5 then limits LATS2 ubiquitination, strengthens Hippo pathway activation, and ultimately inhibits the YAP1/FGFR1 axis, leading to reduced proliferation, migration, and invasion of OC cells.

In in vivo experiments, we established an orthotopic xenograft mouse model of OC and observed that GEM treatment significantly reduced tumor volume and increased the necrosis index. Immunohistochemical analysis further revealed that, compared with the control group, GEM markedly downregulated the expression of HIF1A, UBR5, and FGFR1 in tumor tissues while upregulating LATS2 and p‐YAP1; no notable change was observed in total YAP1 protein levels. These findings suggest that GEM exerts a pronounced antitumor effect in vivo by inhibiting the HIF1A/UBR5 axis, stabilizing LATS2, and activating the Hippo signaling pathway, thereby suppressing the pro‐tumor signaling mediated by YAP1/FGFR1.

In summary, this study elucidated the molecular mechanism by which GEM inhibits OC cell proliferation, migration, and invasion through modulation of the HIF1A/UBR5 axis and the Hippo signaling pathway. These findings provide a new theoretical basis for the clinical application of GEM and identify HIF1A and UBR5 as potential therapeutic targets for developing novel anticancer strategies. This research offers valuable insights into treatment strategies for OC, with significant clinical relevance and application potential. The expression of HIF1A has also been closely linked to GEM resistance in clinical applications. Previous studies have shown that the NF‐κB/AKT signaling pathway regulates HIF1A expression, thereby promoting GEM resistance in lung cancer.^42^ Xu et al. demonstrated that the AKT/HIF1A signaling axis contributes to GEM resistance in pancreatic cancer by modulating glycolytic processes.^43^ Similarly, Luo et al. confirmed that HIF1A influences GEM resistance in triple‐negative breast cancer cells.^44^ In the present study, we elucidated the role of the HIF1A/UBR5 axis in the response of OC to GEM treatment. However, OC is also known for its high level of chemoresistance in clinical settings. Whether the aberrant expression of the HIF1A/UBR5 axis contributes to GEM resistance in OC remains to be further investigated.

The study highlights GEM's potential mechanism of action in OC treatment, particularly its ability to suppress tumor proliferation, migration, and invasion through the HIF1A/UBR5 axis and the Hippo signaling pathway. This discovery provides a fresh theoretical foundation for the clinical use of GEM, especially in patients who do not respond well to conventional chemotherapy. Given the critical roles of HIF1A and UBR5 in tumor angiogenesis and the remodeling of the tumor microenvironment, the results may assist clinicians in tailoring personalized treatment plans based on specific molecular markers. Furthermore, this novel mechanism of GEM action may inspire the development of new drug combinations to enhance its therapeutic efficacy in OC.

Several limitations of the present study warrant consideration. First, current data are insufficient to confirm that the antitumor effect of gemcitabine (GEM) relies predominantly on this single axis. We have not yet systematically evaluated the relative contributions of other potential pathways, such as the DNA damage response, apoptosis pathways, or other Hippo‐independent mechanisms, to the overall efficacy of GEM. Second, although dual‐luciferase reporter assays and ChIP‐PCR confirmed that HIF1A directly drives UBR5 transcription, the context‐dependent variations of this regulatory relationship under different hypoxia levels remain unclear. Future studies are required to systematically delineate how hypoxia gradients modulate the HIF1A–UBR5 signaling axis to further elucidate its biological characteristics. Furthermore, this study did not comprehensively evaluate the potential cytotoxicity of gemcitabine on normal cells, which is crucial for assessing its safety for clinical translation. Although our research focused on HIF1A and UBR5, other critical but unidentified regulatory factors may also be involved in tumor progression, and their specific roles and interplays urgently require further exploration. Additionally, the in vitro concentration of gemcitabine used in this study has not been calibrated against clinically relevant doses. Although previous studies (e.g., PMID: 25184366) have conducted in vitro analyses of gemcitabine, our conclusions still need to be validated in more clinically representative models and patient cohorts.

Moreover, although we performed supplementary prognostic survival analyses, factors related to the HIF1A/UBR5/LATS2 axis did not exhibit significant prognostic correlations within the current cohorts. Therefore, current evidence is insufficient to support this signaling axis as an independent prognostic or predictive biomarker for treatment efficacy. Considering that the prognosis of OC patients is typically influenced by a combination of clinicopathological factors and molecular events, along with inherent issues in public databases—such as sample heterogeneity, incomplete follow‐up information, and inconsistent stratification criteria—these results do not entirely rule out the potential clinical significance of this axis. Nevertheless, its independent prognostic or predictive value must be further verified through future studies incorporating larger sample sizes, complete clinical treatment records, and multivariate regression analyses.

Future research directions should include broader clinical trials to confirm the efficacy and safety of GEM in inhibiting OC via the HIF1A/UBR5 axis and Hippo signaling pathway. Additionally, exploring the potential of combination therapies involving GEM and other anticancer drugs, particularly those targeting the Hippo pathway, could enhance therapeutic effectiveness while minimizing side effects. Further studies on GEM's impact on the tumor microenvironment may reveal additional details of its anticancer mechanisms, facilitating the development of next‐generation therapies. Finally, investigating GEM's potential in treating other cancer types could provide new therapeutic options for a wider range of tumors. Meanwhile, future studies should incorporate larger clinical samples, long‐term follow‐up data, and more systematic univariate and multivariate statistical models to further evaluate the potential application value of the HIF1A/UBR5/LATS2 axis in patient prognostic stratification and treatment response prediction, thereby providing stronger evidence for its clinical translation.

## AUTHOR CONTRIBUTIONS

Jin Zhang conceived the study and designed the methodology; Lixia Xu and Kai Guo conducted the experiments and collected the data; Weijia Dong and Xin Dai performed the formal analysis and visualization; and Donghua Gu supervised the project, acquired funding, and administered the project. All authors contributed to writing, reviewing, and editing the manuscript, and read and approved the final version for publication.

## CONFLICT OF INTEREST STATEMENT

The author declares no conflict of interest.

## ETHICS STATEMENT

All animal experiments were approved by the Animal Ethics Committee of Suzhou Science & Technology Town Hospital (Approval No. SWAE202618).

## Supporting information

Supporting Information S1

## Data Availability

All data generated or analyzed during this study are included in this article and/or its supplementary material files. Further inquiries can be directed to the corresponding author.
