# Supplementary material for: Gemcitabine activates the Hippo signaling pathway and suppresses tumor growth by stabilizing large tumor suppressor kinase 2 through the hypoxia‐inducible factor 1‐alpha/ubiquitin protein ligase E3 component N‐recognin 5 axis
Source: J Cell Commun Signal. 2026 Jun 17;20(2):e70085. doi: 10.1002/ccs3.70085 (PMC13274678; doi:10.1002/ccs3.70085)
Supplement: Supplementary file 1 — Supporting Information S1 [file CCS3-20-e70085-s001.docx]

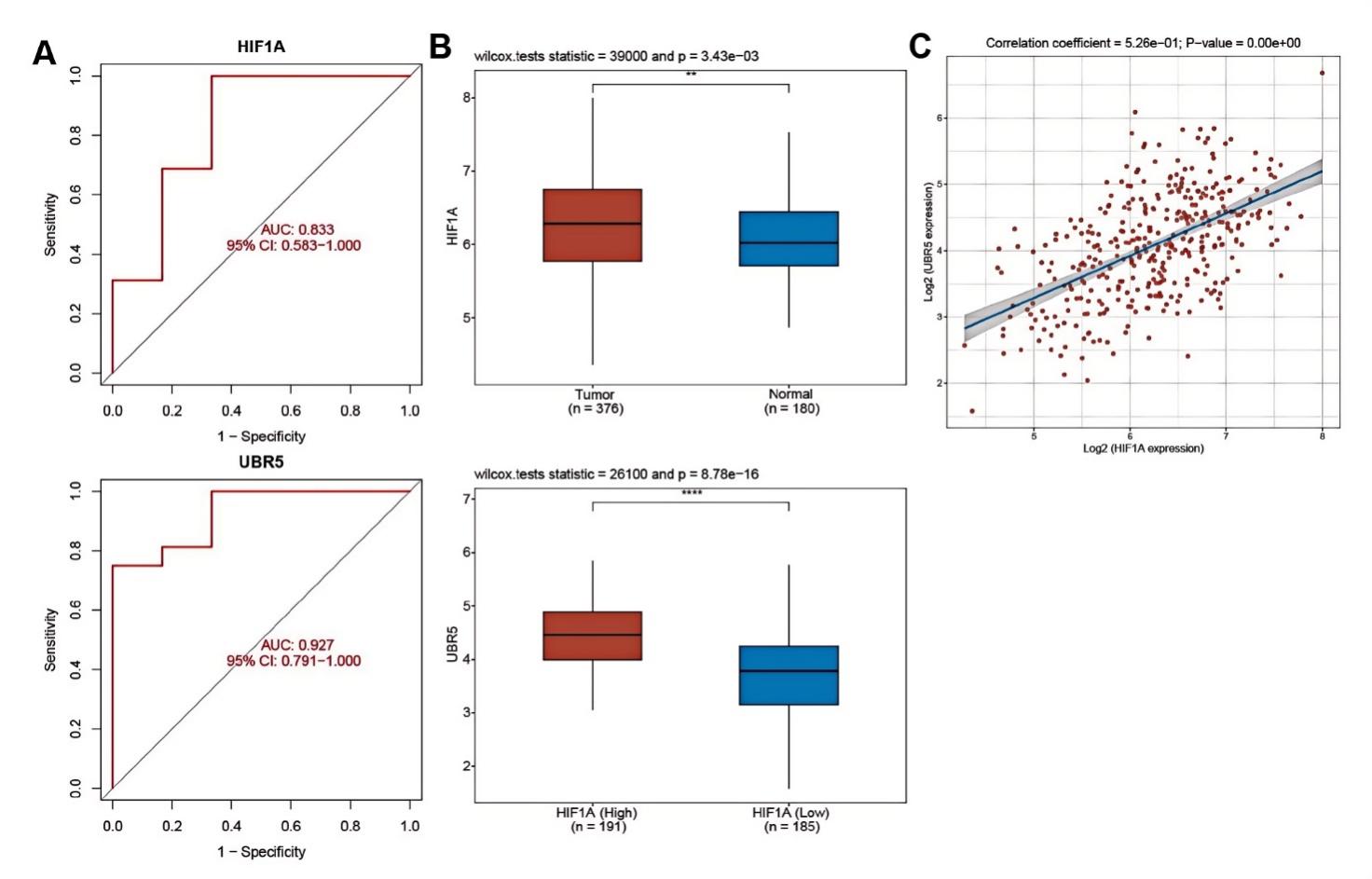


**Figure S1. Differential Expression and Correlation Analysis of HIF1A and UBR5 in OC Tissues.**Note: (A) ROC curve analysis evaluating the diagnostic performance of HIF1A and UBR5 in distinguishing tumor from normal tissues in the GSE54388 dataset. Tumor: n = 16; Normal: n = 6. (B) Wilcoxon rank-sum test assessing HIF1A expression between tumor and normal tissues (top), and UBR5 expression in HIF1A high- vs. low-expression groups (bottom). Tumor: n = 376; Normal: n = 180; HIF1A (High): n = 191; HIF1A (Low): n = 185. (C) Pearson correlation analysis of HIF1A and UBR5 expression levels in tumor tissues (n = 376). * indicates **p* < 0.05; ***p* < 0.01; *****p* < 0.0001.


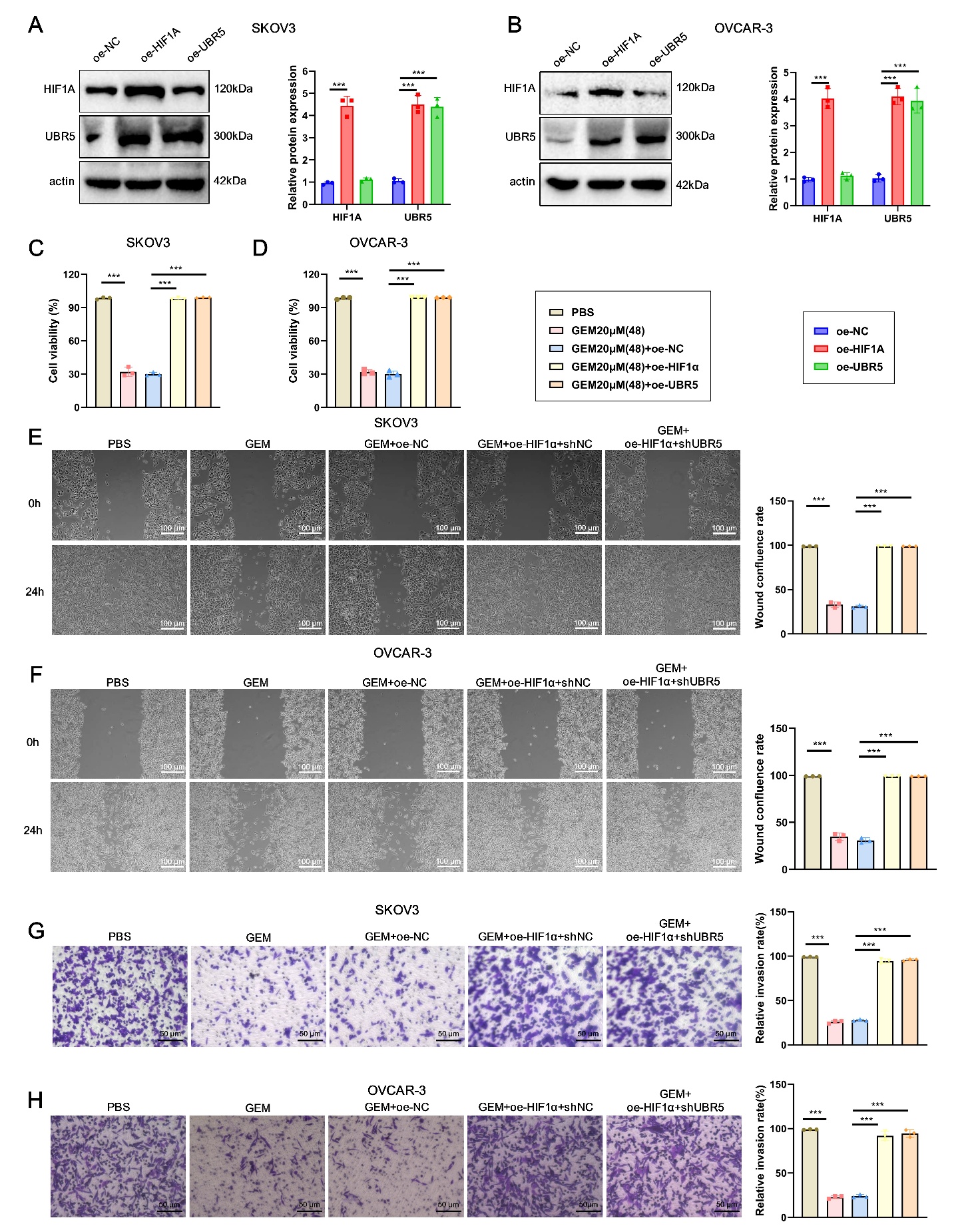


**Figure S2. Effects of GEM on OC Proliferation and Invasion via HIF1A-Mediated UBR5 Regulation.**

Note: (A) WB analysis of HIF1A and UBR5 expression levels under hypoxic conditions in SKOV3 cells; (B) WB analysis of HIF1A and UBR5 expression levels under hypoxic conditions in OVCAR-3 cells; (C) CCK8 assay showing cell viability of SKOV3 cells treated with 20μM GEM for 48 hours; (D) CCK8 assay showing cell viability of OVCAR-3 cells treated with 20μM GEM for 48 hours; (E) Wound healing assay assessing the migration rate of SKOV3 cells treated with 20μM GEM for 48 hours (Scale bar: 100μm); (F) Wound healing assay assessing the migration rate of OVCAR-3 cells treated with 20μM GEM for 48 hours (Scale bar: 100μm); (G) Transwell invasion assay showing the invasive capacity of SKOV3 cells treated with 20μM GEM for 48 hours (Scale bar: 50μm); (H) Transwell invasion assay showing the invasive capacity of OVCAR-3 cells treated with 20μM GEM for 48 hours (Scale bar: 50μm). Results are presented as Mean ± SD. Cell experiments were repeated three times. ****p* < 0.001, statistical analysis was performed using ANOVA and Tukey's multiple comparison test.


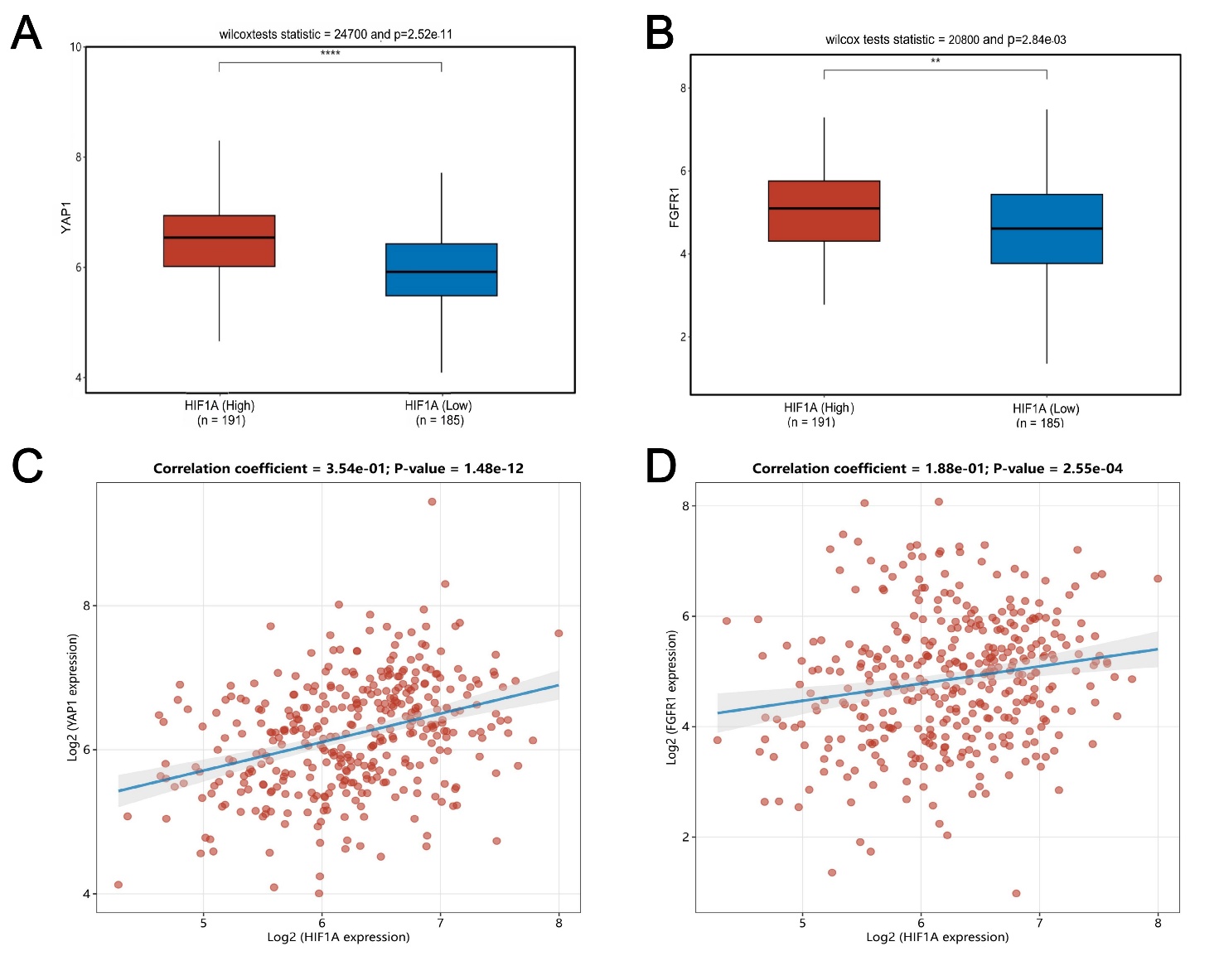


**Figure S3. Correlation analysis of HIF1A with YAP1 and FGFR1 in ovarian cancer tissues.**

Note: (A-B) Wilcoxon rank-sum test analysis of YAP1 (A) and FGFR1 (B) expression levels in HIF1A high- and low-expression groups, HIF1A (High): n = 191; HIF1A (Low): n = 185; (C-D) Pearson correlation analysis of HIF1A expression with YAP1 (C) and FGFR1 (D), Tumor: n = 376. ** indicates comparison between two groups, *p* < 0.01; *****p* < 0.00001.
